# Disordered but effective: short linear motifs as gene therapy targets for hyperexcitability disorders

**DOI:** 10.1172/JCI182198

**Published:** 2024-07-01

**Authors:** Sulayman D. Dib-Hajj, Stephen G. Waxman

**Affiliations:** 1 Department of Neurology and; 2 Center for Neuroscience & Regeneration Research, Yale University, New Haven, Connecticut, USA.; 3 Center for Rehabilitation Research, VA Connecticut Healthcare System, West Haven, Connecticut, USA.

## Abstract

Multiple approaches have targeted voltage-gated sodium (Nav) channels for analgesia. In this issue of the *JCI*, Shin et al. identified a peptide aptamer, Na_V_iPA1, carrying a short polybasic motif flanked by serine residues in a structurally disordered region of loop 1 in tetrodotoxin-sensitive (TTX-S) but not tetrodotoxin-resistant (TTX-R) channels. Na_V_iPA1h inhibited TTX-S Na_V_ channels and attenuated excitability of sensory neurons. Delivery of Na_V_iPA1 in vivo via adeno-associated virions restricted its expression to peripheral sensory neurons and induced analgesia in rats. Targeting of short linear motifs in this manner may provide a gene therapy modality, with minimal side effects due to its peripherally-restricted biodistribution, which opens up a therapeutic strategy for hyperexcitability disorders, including pain.

## Na_V_ channels as targets for pain disorders

**Figure 1:**
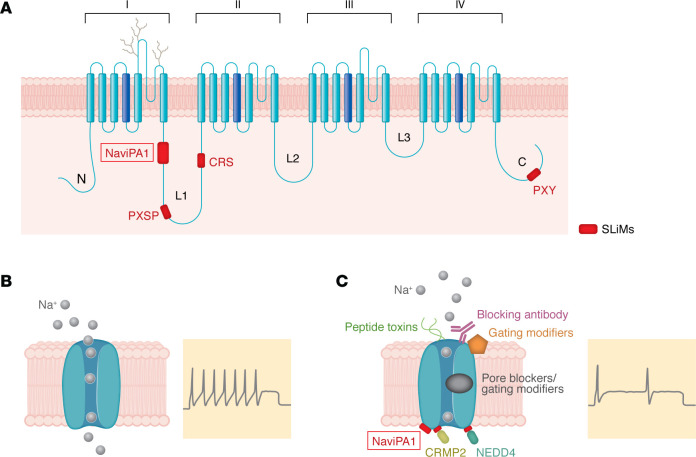
Sodium channel structure offers multiple inhibitory modalities to treat excitability disorders including pain. (**A**) The pore-forming α-subunit of sodium channels has 24 transmembrane segments, organized into four domains (I, II, III, and IV), linked by three cytoplasmic loops (L1–3), with a cytoplasmic N- and C-termini of the polypeptide. The cytoplasmic regions of the TTX-S channels carry SLiMs, including sites for posttranslational modifications, e.g. p38 MAPK phosphorylation (PXSP), binding channel partners that regulate channel trafficking and reduce number of channels at the cell surface (CRMP2 Regulatory Sequence, CRS; PXY, which binds NEDD4 family of E3 ubiquitin ligases), and binding of NaviPA1, which reduces current density of multiple TTX-S channels, albeit via an unknown mechanism. (**B**) TTX-S Nav channels contribute to hyperexcitability of sensory neurons as reflected by repetitive action potential firing. (**C**) There are multiple strategies for targeting Nav channels, including those mediated by SLiMs, (e.g., via CRMP2, NEDD4, and NaviPA1). Other strategies include small molecule inhibitors that reduce the amplitude of the Nav current by blocking the channel pore (e.g., TTX and its derivatives), while others also stabilize inactivated states of the channel (e.g., local anesthetics). Peptide toxins can also act as pore blockers, and others bind to the VSM and modulate gating properties. Biologics like antibodies and nanobodies that target channels at the cell surface provide another possible approach, albeit without reportable success to date. Inhibition of Nav channels by these different modalities attenuates firing of sensitized neurons leading to analgesia.

Voltage-gated sodium (Na_V_) channels are obligate components of the electrogenic machinery that underlie action potential electrogenesis (1). Nine members of the pore-forming α-subunit in the mammalian Na_V_ channel family, expressed in a tissue-specific manner, have distinct biophysical properties and can be grouped into two pharmacologically distinct classes, as tetrodotoxin sensitive (TTX-S) and resistant (TTX-R) (2). The Nav α subunit (Figure 1A) consists of four homologous domains (DI–DIV), each with six transmembrane segments (S1–S6) that are functionally organized into a voltage-sensing module (S1–S4), and a pore module (S5 and S6 and P-loop), with cytoplasmic N- and C-termini and loops that join the four domains (L1–L3) (3). The sequences of the transmembrane segments and L3 are highly conserved, whereas the N- and C-terminus and L1 and L2 are more diverse in length and primary sequence, which suggests that these parts of the channel may contribute to differential distribution in neuronal compartments and impart distinct biophysical properties on channel isoforms.

Primary sensory neurons are responsible for the transduction of environmental stimuli and the transmission of the signal to the first synapse in the dorsal horn of the spinal cord, and their sensitization contributes to the induction and maintenance of chronic pain (4). Adult mammalian sensory neurons in dorsal root ganglion (DRG) and trigeminal ganglion express multiple TTX-S (Na_V_1.1, Na_V_1.6, Na_V_1.7) and TTX-R (Na_V_1.8, Na_V_1.9) channel isoforms (5, 6); the TTX-S Na_V_1.3 channel is expressed during embryonic development and reemerges following injury (7). Human and animal studies have confirmed that an increase in Na_V_ channel levels or gain-of-function in biophysical properties lead to neuronal hyperexcitability (Figure 1B), which contributes to peripheral sensory neuron sensitization leading to pain (5, 6), supporting the idea that these channels are opportune targets for the development of therapeutic strategies for pain disorders.

While multiple nonselective Na_V_ channel pore-blocker inhibitors, which attenuate neuronal firing (Figure 1C), are routinely used for treatment of another hyperexcitability disorder, epilepsy, fewer have been successfully used for the treatment of painful disorders such as trigeminal neuralgia, albeit with side effects that can lead to treatment withdrawal (8), and are not among those recommended for first line treatment of neuropathic pain (9). This dearth has resulted in efforts to target Na_V_ channels that are preferentially expressed in peripheral neurons and may yield analgesics with minimal side effects. Chief among these targets has been the Na_V_1.7 channel because of strong genetic validation in humans; gain-of-function mutations cause painful disorders while loss-of-function mutations cause loss of pain (5, 6). However, small molecule inhibitors of Na_V_1.7, which bind to the voltage sensor module (VSM) and induce state-dependent inhibition of the channel, or nonselective pore blockers (Figure 1C), have not cleared clinical testing, possibly due to limited target engagement in vivo (8) and/or to autonomic symptoms from on-target effects on Na_V_1.7 in sympathetic and parasympathetic neurons and baroreceptors (10, 11). It is notable that subjects with Nav1.7-related congenital insensitivity to pain and those on long-term treatment with pan sodium channel blockers do not report these autonomic deficits (12), which suggests that development of safer Nav1.7 blockers might be possible, as the basis of the sparing of autonomic function in these subjects becomes better understood. Recently, a selective small molecule blocker of Na_V_1.8, VX-548, has been shown to produce partial pain relief in Phase 3 clinical trials of acute pain (13, 14). A CNS-penetrant aryl sulfonamide small molecule Na_V_1.6 selective blocker (15) is in a Phase 2 clinical trial as an adjunctive therapy for seizures produced by mutations of *SCN8A* (the gene encoding Nav1.6), but has not yet been tested in pain conditions; on-target side effects will have to be monitored closely because of the channel’s widespread expression in the CNS and PNS neurons. It remains to be seen whether small molecules can achieve sufficient inhibition of single Na_V_ channel isoforms to provide effective monotherapies for chronic pain or whether multi-channel blockade is required for effective analgesia.

## Strategies for reducing Nav channel expression

Among alternative strategies, preclinical studies involving multiple gene therapy and biologic approaches that knockdown expression of Na_V_ channels include AAV-mediated delivery of shRNA against Nav1.3 (16), Nav1.7 (17), and CRISPR-dCas9 and zinc finger protein suppression of expression of Nav1.7 (18). These approaches have produced effective analgesia in animal models of pain. Antibodies and venom peptides present another platform to develop Na_V_ isoform–selective therapeutics (Figure 1C) (19).

An alternative approach to target Na_V_ channels for analgesia with a different strategy aims at reducing channel density at the plasma membrane. The cytoplasmic regions of Na_V_ channels are hubs for signaling complexes that carry short linear motifs (SLiMs) and are suggested to mediate protein-protein interactions that regulate channel trafficking to, and stability at, the plasma membranes (20–22). SLiMS in the L1 and C-terminus of multiple Na_V_ channels have been shown to regulate Na_V_ current density. Phosphorylation of a specific serine-proline dipeptide in L1 of Na_V_1.6 by p38 MAPK recruits NEDD4-2 ubiquitin ligase to the channel and induces its internalization (23). The PXY motif in the C-terminus of multiple Na_V_ channels also recruits NEDD4-2 ubiquitin ligases and induces their internalization (22). Activation of p38 MAPK by treatment of DRG neurons with TNF-α causes phosphorylation of a SP dipeptide in the N-terminus of Na_V_1.7, which alters insertion of channels in the plasma membrane (24). Another example of the modulation of channel density is provided by the observation that treatment of DRG neurons with anisomycin causes an increase in Na_V_1.8 current density in a p38 MAPK dependent manner, due to the phosphorylation of SP dipeptides in the L1 of the channel (25). The SP phosphoacceptor sites in Na_V_1.6, Na_V_1.7, and Na_V_1.8 are part of PXSP motifs suggestive of a molecular switch for protein-protein interactions that regulates channel levels in the plasma membrane. A different short sequence motif in L1 of Na_V_1.7 channels was identified as the site of CRMP2 binding (CRMP2 regulatory sequence, CRS) and administering CRS via a cell-permeable peptide or via an AAV9 capsid–caused channel internalization, thereby reducing DRG neuron excitability and ameliorating pain in a mouse model of neuropathic pain (26). Thus, the cytoplasmic regions of Na_V_ channels provide a plethora of molecular targets for the development of treatment strategies.

## Targeting Nav1.7 with a peptide aptamer

In a well-designed study in this issue, Shin and colleagues (27) used in silico analysis of intrinsically disordered domains (IDD) in cytoplasmic sequences of Na_V_1.7 channel and identified a polyampholytic interfering peptide aptamer (which they called Na_V_iPA1) that corresponded to a sequence in the proximal end of L1 (Figure 1A). The Na_V_iPA1 sequence carries a short linear polybasic motif and potentially phosphorylatable serine residues, which are conserved in TTX-S but not TTX-R channels. Functional testing showed that Na_V_iPA1 suppressed TTX-S sodium currents and excitability of DRG neurons when delivered via an adeno associated viral vector (AAV6- Na_V_iPA1), leading to analgesia in a rat model of neuropathic pain. IDD protein modules have been proposed as critical in cellular signaling pathways, especially because they can assume different conformations upon phosphorylation, and possibly other posttranslational modifications, and thus act as molecular switches that impact function (28). The inhibitory effect of Na_V_iPA1 on endogenous TTX-S currents, but not TTX-R, BK potassium channels, or high-voltage activated calcium channels was confirmed in rat DRG neurons and neurons differentiated from human induced pluripotent stem cells. Biochemical assays suggested that Na_V_iPA1 did not interfere with channel intracellular trafficking, but the data were consistent with NaViPA1 binding to the channel protein and exerting an effect via an intramolecular domain-domain interaction. The inhibitory effect on channels was dependent on the polybasic motif and a subset of serine residues within Na_V_iPA1. Together, these results indicate that an AAV-mediated peripherally restricted iPA can block multiple Na_V_ channels in sensory neurons and reduce pain in animal models, suggesting that targeting of SLiMs may provide an approach in the treatment of hyperexcitability disorders such as pain and epilepsy.

The Shin et al. study (27) leaves some questions unanswered and also suggests new opportunities. It is not known whether the SLiMs in the Na_V_iPA1 exert their effects as a decoy via an adapter molecule or via an intramolecular interaction; and the lack of high-resolution atomic structures of cytoplasmic regions of Na_V_ channels published to date limits our ability to predict and test potential intramolecular interacting sequences that might influence channel trafficking. We can speculate that the inhibitory effect of Na_V_iPA1 suggests that the orthogonal polybasic motif and the subset of serine residues in the full-length Na_V_ channels might induce autoinhibition to maintain neuronal homeostasis and reduce potential activity-dependent injury to neurons. However, a recent study showed that alanine substitution of this polybasic motif in Na_V_1.7 did not alter the channel’s delivery and distribution in soma and axons of sensory neurons (29). Thus, the physiological function of this polybasic motif under normal conditions, or conditions when it might induce an inhibitory effect, remain to be determined.

Another unexpected finding was the nuclear localization of Na_V_iPA1 driven by a putative nuclear localization signal contained within the polybasic motif. There is currently no evidence that the channel itself can relocate to the nucleus or that the L1 of TTX-S channels is cleaved under physiological or pathological conditions and translocated to the nucleus, where it might affect gene expression or other aspects of nuclear function. Localization and aggregation of Na_V_iPA1 in the nucleus raises a concern about potential off-target toxicity effects, especially upon long-term treatment, which is required in chronic pain conditions. Finally, while Na_V_iPA1 has been confirmed to spare BK potassium channels and high-voltage activated calcium channels, determining off-target effects on other ion channels, receptors, and signaling complexes that regulate neuronal excitability will be a challenge because it is difficult to find a neuronal testing system devoid of TTX-S channels and it is impractical to test the myriad channels and receptors individually in heterologous expression systems.

## Conclusions

The study by Shin et al. (27) provides an example of an analgesic gene therapy approach that reduces proexcitatory TTX-S sodium currents in peripheral sensory neurons by roughly 50%, which also adds to our foundational knowledge of the role of specific SLiMs within cytoplasmic regions of Na_V_ channels in regulating channel’s biophysical properties, delivery, and stability at the plasma membrane. Although the physiological role of the IDD regions of Na_V_ channels are not well understood and the mechanism underlying the inhibitory effects of Na_V_iPA1 on the TTX-S channels remain to be elucidated, targeting of protein-protein interacting sites may offer an innovative strategy for dissecting structure-function models of Na_V_ channels. In addition, of course, this approach may provide a route to development of effective treatments for pain. Further, since Na_V_iPA1 acts on all of the neuronal TTX-S Na_V_ channels, this approach might be used to treat other excitability disorders, for example epilepsy. It remains to be seen whether a clinical version of the Na_V_iPA1 will prove to be an effective analgesic in humans, especially if it avoids adverse autonomic and motor effects, but the Shin et al. (27) preclinical studies provide a first step.

## Version 1 07/01/2024

Electronic publication

## References

[1] Hodgkin AL, Huxley AF (1952). A quantitative description of membrane current and its application to conduction and excitation in nerve. J Physiol.

[2] Catterall WA (2005). International union of pharmacology. XLVII. Nomenclature and structure-function relationships of voltage-gated sodium channels. Pharmacol Rev.

[3] Catterall WA (2020). The conformational cycle of a prototypical voltage-gated sodium channel. Nat Chem Biol.

[4] Basbaum AI (2009). Cellular and molecular mechanisms of pain. Cell.

[5] Bennett DL (2019). The role of voltage-gated sodium channels in pain signaling. Physiol Rev.

[6] Dib-Hajj SD, Waxman SG (2019). Sodium channels in human pain disorders: genetics and pharmacogenomics. Annu Rev Neurosci.

[7] Waxman SG (1994). Type III sodium channel mRNA is expressed in embryonic but not adult spinal sensory neurons, and is reexpressed following axotomy. J Neurophysiol.

[8] Alsaloum M (2020). Status of peripheral sodium channel blockers for non-addictive pain treatment. Nat Rev Neurol.

[9] Finnerup NB (2015). Pharmacotherapy for neuropathic pain in adults: a systematic review and meta-analysis. Lancet Neurol.

[10] Regan CP (2024). Autonomic dysfunction linked to inhibition of the Na_v_1.7 sodium channel. Circulation.

[11] Deng L (2023). Nav1.7 is essential for nociceptor action potentials in the mouse in a manner independent of endogenous opioids. Neuron.

[12] Waxman SG, Dib-Hajj SD (2023). Na_V_1.7: a central role in pain. Neuron.

[13] Jones J (2023). Selective inhibition of Na_V_1.8 with VX-548 for acute pain. N Engl J Med.

[14] Waxman SG (2023). Targeting a peripheral sodium channel to treat pain. N Engl J Med.

[15] Johnson JP (2022). NBI-921352, a first-in-class, NaV1.6 selective, sodium channel inhibitor that prevents seizures in Scn8a gain-of-function mice, and wild-type mice and rats. Elife.

[16] Samad OA (2013). Virus-mediated shRNA knockdown of Na(v)1.3 in rat dorsal root ganglion attenuates nerve injury-induced neuropathic pain. Mol Ther.

[17] Cai W (2016). shRNA mediated knockdown of Nav1.7 in rat dorsal root ganglion attenuates pain following burn injury. BMC Anesthesiol.

[18] Moreno AM (2021). Long-lasting analgesia via targeted in situ repression of NaV1.7 in mice. Sci Transl Med.

[19] Wulff H (2019). Antibodies and venom peptides: new modalities for ion channels. Nat Rev Drug Discov.

[20] Cheng X (2021). Mini-review - sodium channels and beyond in peripheral nerve disease: modulation by cytokines and their effector protein kinases. Neurosci Lett.

[21] Dib-Hajj SD, Waxman SG (2010). Isoform-specific and pan-channel partners regulate trafficking and plasma membrane stability; and alter sodium channel gating properties. Neurosci Lett.

[22] Laedermann CJ (2015). Post-translational modifications of voltage-gated sodium channels in chronic pain syndromes. Front Pharmacol.

[23] Gasser A (2010). Two Nedd4-binding motifs underlie modulation of sodium channel Nav1.6 by p38 MAPK. J Biol Chem.

[24] Tyagi S (2024). Compartment-specific regulation of Na_V_1.7 in sensory neurons after acute exposure to TNF-α. Cell Rep.

[25] Hudmon A (2008). Phosphorylation of sodium channel Na(v)1.8 by p38 mitogen-activated protein kinase increases current density in dorsal root ganglion neurons. J Neurosci.

[26] Gomez K (2023). Identification and targeting of a unique Na_V_1.7 domain driving chronic pain. Proc Natl Acad Sci U S A.

[27] Shin SM (2024). Peripherally targeted analgesia via AAV-mediated sensory neuron-specific inhibition of multiple pronociceptive sodium channels in rat. J Clin Invest.

[28] Wright PE, Dyson HJ (2015). Intrinsically disordered proteins in cellular signalling and regulation. Nat Rev Mol Cell Biol.

[29] Tyagi S (2023). Conserved but not critical: trafficking and function of Na(V)1.7 are independent of highly conserved polybasic motifs. Front Mol Neurosci.

